# Retrospective analysis of interpretable machine learning in predicting ICU thrombocytopenia in geriatric ICU patients

**DOI:** 10.1038/s41598-024-67785-1

**Published:** 2024-07-20

**Authors:** Yingting Xu, Weimin Zhang, Xuchao Ma, Muying Wu, Xuandong Jiang

**Affiliations:** Intensive Care Unit, Affiliated Dongyang Hospital of Wenzhou Medical University, No. 60 Wuning West Road, Dongyang, Jinhua, Zhejiang People’s Republic of China

**Keywords:** Geriatric patients, ICU, Postoperative, Thrombocytopenia, Machine learning, Predictive modeling, Risk factors, Haematological diseases, Biomarkers, Translational research

## Abstract

We developed an interpretable machine learning algorithm that prospectively predicts the risk of thrombocytopenia in older critically ill patients during their stay in the intensive care unit (ICU), ultimately aiding clinical decision-making and improving patient care. Data from 2286 geriatric patients who underwent surgery and were admitted to the ICU of Dongyang People’s Hospital between 2012 and 2021 were retrospectively analyzed. Integrated algorithms were developed, and four machine-learning algorithms were used. Selected characteristics included common demographic data, biochemical indicators, and vital signs. Eight key variables were selected using the Least Absolute Shrinkage and Selection Operator and Random Forest Algorithm. Thrombocytopenia occurred in 18.2% of postoperative geriatric patients, with a higher mortality rate. The C5.0 model showed the best performance, with an area under the receiver operating characteristic curve close to 0.85, along with unparalleled accuracy, precision, specificity, recall, and balanced accuracy scores of 0.88, 0.98, 0.89, 0.98, and 0.85, respectively. The support vector machine model excelled at predictively assessing thrombocytopenia severity, demonstrating an accuracy rate of 0.80 in the MIMIC database. Thus, our machine learning-based models have considerable potential in effectively predicting the risk and severity of postoperative thrombocytopenia in geriatric ICU patients for better clinical decision-making and patient care.

## Introduction

With a rapidly aging population worldwide, the global population aged ≥ 65 years is projected to reach 973 million by 2030, a change that implies a growing proportion of older persons in the healthcare system and a simultaneous increase in their need for postoperative intensive care unit (ICU) monitoring^1–4^. The gradual deterioration of physiological function and the accumulation of multiple chronic diseases in geriatric ICU patients make them more prone to various complications after undergoing surgical treatment. Of these, thrombocytopenia is a common perioperative complication, with a prevalence of 5–10%, which may be higher in critically ill geriatric patients^5^. Platelets are not only involved in hemostasis but also in inflammatory and immune responses and wound healing, leading to an increased risk of bleeding, a higher rate of transfusion, and a significantly increased risk of death in patients with thrombocytopenia^6,7^. Therefore, the early prediction and identification of the risk of thrombocytopenia is particularly important.

With advances in medical research and technology, the use of big data in healthcare also offers opportunities for early prediction and management of thrombocytopenia. Large amounts of data from patients are collected and stored, providing a valuable resource. However, conventional methods are unsuited for large amounts of medical data that are increasing in complexity. Machine learning, an advanced data analytics method that captures complex patterns in data to provide more accurate predictions, has been widely used in medical research^8–10^. Moreover, to optimize the application of machine learning models, various tools and methods have been developed to identify and visualize key variables that are determinative for predicting outcomes and to visualize them to provide intuitive and powerful support for clinical decision-making. For example, Tseng et al*.* utilized an AI-based machine learning approach to predict cardiac surgery-associated acute kidney injury through perioperative data-driven learning and achieved good predictive results and model explanation using the SHapley Additive exPlanation (SHAP) method^11^. Caicedo-Torres et al*.* proposed a novel and visually interpretable multi-scale convolutional architecture for predicting intra-ICU mortality in patients^12^.

Despite the successful application of machine learning in other areas of medicine, there is a lack of research on the prediction of postoperative thrombocytopenia in critically ill geriatric patients. Therefore, this research aimed to employ several machine learning techniques to simulate and explicate the risk of thrombocytopenia in older ICU patients post-surgery and develop corresponding predictive tools to estimate its probability. The models were subsequently validated utilizing the publicly accessible MIMIC dataset^13^.

## Methods

### Study design

This study was conducted in compliance with the Transparent Reporting of a multivariable prediction model for Individual Prognosis or Diagnosis (TRIPOD) Statement (Additional File: Table S1)^14^. Data from 2286 postoperative geriatric ICU patients hospitalized at Dongyang People’s Hospital between 2012 and 2021 were retrospectively analyzed. The inclusion criteria were as follows: duration of ICU stay > 24 h, first admission to ICU, and age ≥ 65 years. The exclusion criteria were as follows: patients with hematologic malignancies, cirrhosis, or prior splenectomy; those who presented with thrombocytopenia prior to ICU admission; those with heparin-induced thrombocytopenia (HIT); and those with > 30% of data missing. The publicly available MIMIC database was used for external validation.

This study adhered to all relevant local guidelines and regulations and was approved by the Ethics Committee of Dongyang People’s Hospital (DRY-2022-YX-155). Due to the retrospective and observational nature of the study, the requirements of informed consent were waived by the ethics committee of Dongyang People’s Hospital. Before conducting the analysis, the data were anonymized. After completing the online training course, “Protecting Human Research Participants”, offered by the National Institutes of Health (Certification number 7632299), author XJ was granted permission to access the MIMIC-III database.

### Data collection

Data collection was performed using case information mining software by Shanghai LE9 Health Co., Ltd. The data collected included age; sex; Sequential Organ Failure Assessment (SOFA) score; Glasgow Coma Scale score; history of smoking; history of alcohol use disorder; comorbidities such as hypertension and diabetes mellitus; types of vasopressors, glucocorticoids, and antiplatelets used during the first 24 h after ICU admission; initial biochemical indicators; and maximum, minimum and mean vital signs on ICU admission.

The primary outcome was in-hospital mortality. Secondary outcomes included duration of mechanical ventilation, duration of ICU stay, duration of hospitalization, and hospitalization costs.

#### Diagnostic criteria

Thrombocytopenia was defined as platelet count < 100 × 10^9^/L during the ICU stay. Thrombocytopenia was further categorized into mild, moderate, and severe, defined as platelet counts < 100 × 10^9^/L, < 50 × 10^9^/L, and < 20 × 10^9^/L, respectively^15,16^.

### Data processing

The primary aim of this study was to accurately predict the occurrence of thrombocytopenia, defined as a platelet count less than 100 × 10^9^/L, among postoperative geriatric ICU patients and to facilitate its direct application in a clinical setting utilizing a binary classification approach (Yes/No) through our machine learning model, ultimately supporting timely and effective interventions. A preliminary screening of 87 potentially relevant variables was conducted. The Classification and REgression Training (CARET) R package was used for data preprocessing. Seven variables with strong correlation (correlation coefficient > 0.9) were eliminated, and the remaining 80 variables were screened using Least Absolute Shrinkage and Selection Operator regression. The cv.glmnet function was used to select the Least Absolute Shrinkage and Selection Operator regularization parameter lambda through tenfold cross-validation, and variables were progressively selected by controlling the value of lambda^17^. Further, the random forest method was utilized for variable selection. Finally, eight variables were included: initial platelet count at ICU admission, creatinine, hemoglobin, glutamic oxalacetic transaminase (AST), minimum and maximum systolic blood pressure, and mean and maximum heart rate within 24 h of ICU admission.

The interquartile range, namely the distance between the upper and lower quartiles of the box plot, was used to detect outliers. Outliers were excluded and treated as missing values. Variables with more than 30% of values missing were removed. The multiple imputation method was used to replace missing variable values^18,19^. Specifically, an imputation model was designated for each incomplete variable, using other variables as predictors. In each iteration, the variables obtained from the imputation were subsequently used in the subsequent imputation models, and this process was repeated until convergence was achieved.

### Model construction

First, the CARET, XGBoost, C50, e1071, naivebayes, and gbm R packages were used to train four machine learning (ML) models with support vector machine (SVM), Bayesian machine learning, C5.0 decision tree, and eXtreme Gradient Boosting (XGBoost) algorithms. Hyperparameters were adjusted by grid search. The four models were then used to create an integrated model using CaretEnsemble^20^. Samples were randomly divided into a training set and a validation set at a 7:3 ratio. All ML models were evaluated using 10× cross-validation. The MIMIC dataset was used for external validation.

### Model validation and evaluation

Model performance was evaluated with the area under the receiver operating characteristic curve (AUC). The calibration performance of the models was evaluated using calibration curves, while the confusion matrix was evaluated using accuracy, precision, specificity, and recall as parameters, and the cut-off point was 0.5.

### Model explanation and application

The model explanation was accomplished using variable importance, which was ranked using the “varImp” function in the CARET R package. Moreover, individual explanations were provided by the locally interpretable model-agnostic explanation (LIME) and iBreakdown algorithms^21,22^. Finally, a web-based calculator based on the optimal C5.0 algorithm model was developed, which enabled the calculation of the probability of thrombocytopenia during ICU stay in an individual patient by entering 8 parameters: initial platelet count on ICU admission, creatinine, hemoglobin, and AST, as well as minimum and maximum systolic blood pressure and mean and maximum heart rate within 24 h after ICU admission (Additional File: Fig. S1). This tool was uploaded to GitHub and made publicly available. Additionally, to differentiate the severity of platelet decline, the same method was used to screen eight variables: SOFA score, platelet count, arterial pH, serum lactate international normalized ratio, serum sodium level, minimum systolic blood pressure, and norepinephrine usage. A prognostic model was constructed employing the SVM algorithm, incorporating these determinants. Correspondingly, a web-based calculator was formulated based on this model and is accessible via the supplementary materials, denoted as Fig. S2.

### Statistics

Descriptive statistics were analyzed using the CBCgrps R package^23^. Measures conforming to the normal distribution are expressed as x ± s, and measures not conforming to the normal distribution are expressed as M (P25, P75). Analysis of variance was used to compare continuous variables, and the Chi-squared test was used to compare categorical variables. All statistical analyses were performed using R software (version 4.1.3 (https://mirrors.tuna.tsinghua.edu.cn/CRAN/)). Statistical significance was set at *P* < 0.05.

## Results

### Study population

**Figure 1:**
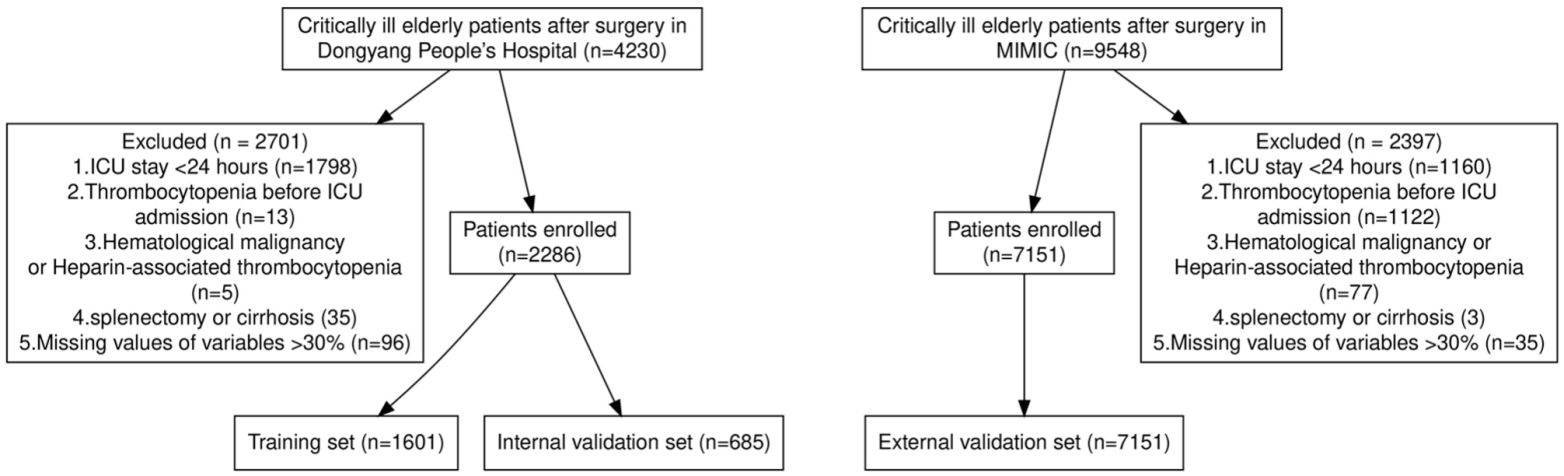
Flow chart of the study. *ICU* intensive care unit.

The study flowchart is shown in Fig. 1. After 3526 patients were excluded, 2286 patients were ultimately included. Patients were randomly divided into a training set and an internal validation set at a 7:3 ratio. MIMIC iii was used for external validation. A comparison of the baseline data between the training set, the internal validation set, and the external validation set is shown in Table 1.

**Table 1:** Feature distribution comparison: training, internal validation, and external validation.

Variables	Training (n = 1601)	Internal validation (n = 685)	External validation (n = 7151)	P value
Age (years), mean (SD)	75.8 ± 7.6	76.3 ± 7.3	76.9 ± 7.2	< 0.001
Male, n (%)	858 (53.6)	366 (53.4)	3943 (55.1)	0.409
SOFA, mean (SD)	5 ± 3.1	4.9 ± 3.1	4.3 ± 2.5	< 0.001
Hypertension, n (%)	857 (53.5)	353 (51.5)	4288 (60)	< 0.001
Diabetes, n (%)	224 (14)	89 (13)	2024 (28.3)	< 0.001
Vasopressor used, n (%)	640 (40)	273 (39.9)	1300 (18.2)	< 0.001
Platelet (× 10^9^/L), mean (SD)	165.5 ± 65	162.5 ± 64.4	196.7 ± 80.3	< 0.001
Hemoglobin (g/L), mean (SD)	108.4 ± 19.5	107.7 ± 19.5	104.7 ± 17.9	< 0.001
Creatinine (mmol/L), mean (SD)	80.7 ± 40.8	77.2 ± 37.2	91 ± 48.8	< 0.001
Glutamic oxalacetic transaminase (U/L), median (IQR)	32 (22, 62)	31 (23, 55)	35 (23, 67)	0.008
Minimum systolic pressure (mmHg), mean (SD)	97 ± 17.1	97.7 ± 16.3	89.5 ± 15.7	< 0.001
Maximum systolic pressure (mmHg), mean (SD)	170.6 ± 26.3	173.9 ± 27	154.9 ± 20.6	< 0.001
Average heartrate (bpm), mean (SD)	82.9 ± 14.7	82.4 ± 14.6	82.4 ± 12	0.436
Maximum heart rate (bpm), mean (SD)	106.2 ± 21.7	106.3 ± 21.4	99.9 ± 16.7	< 0.001
Thrombocytopenia, n (%)	293 (18.3)	119 (17.4)	1443 (20.2)	0.069
Hospital mortality, n (%)	183 (11.4)	77 (11.2)	864 (12.1)	0.655
ICU length of stay (days), median (IQR)	3.6 (1.8, 7.8)	2.9 (1.8, 7.7)	2.5 (1.5, 4.9)	< 0.001
Ventilation duration (days), median (IQR)	0.8 (0.2, 3.8)	0.8 (0.2, 3.9)	6.17 (0, 21.4)	< 0.001
Hospital length of stay (days), median (IQR)	21 (14, 30)	20 (14, 30)	8.1 (5.5, 12.8)	< 0.001

*ICU* intensive care unit, *SOFA* sepsis-related organ failure assessment.

**Figure 2:**
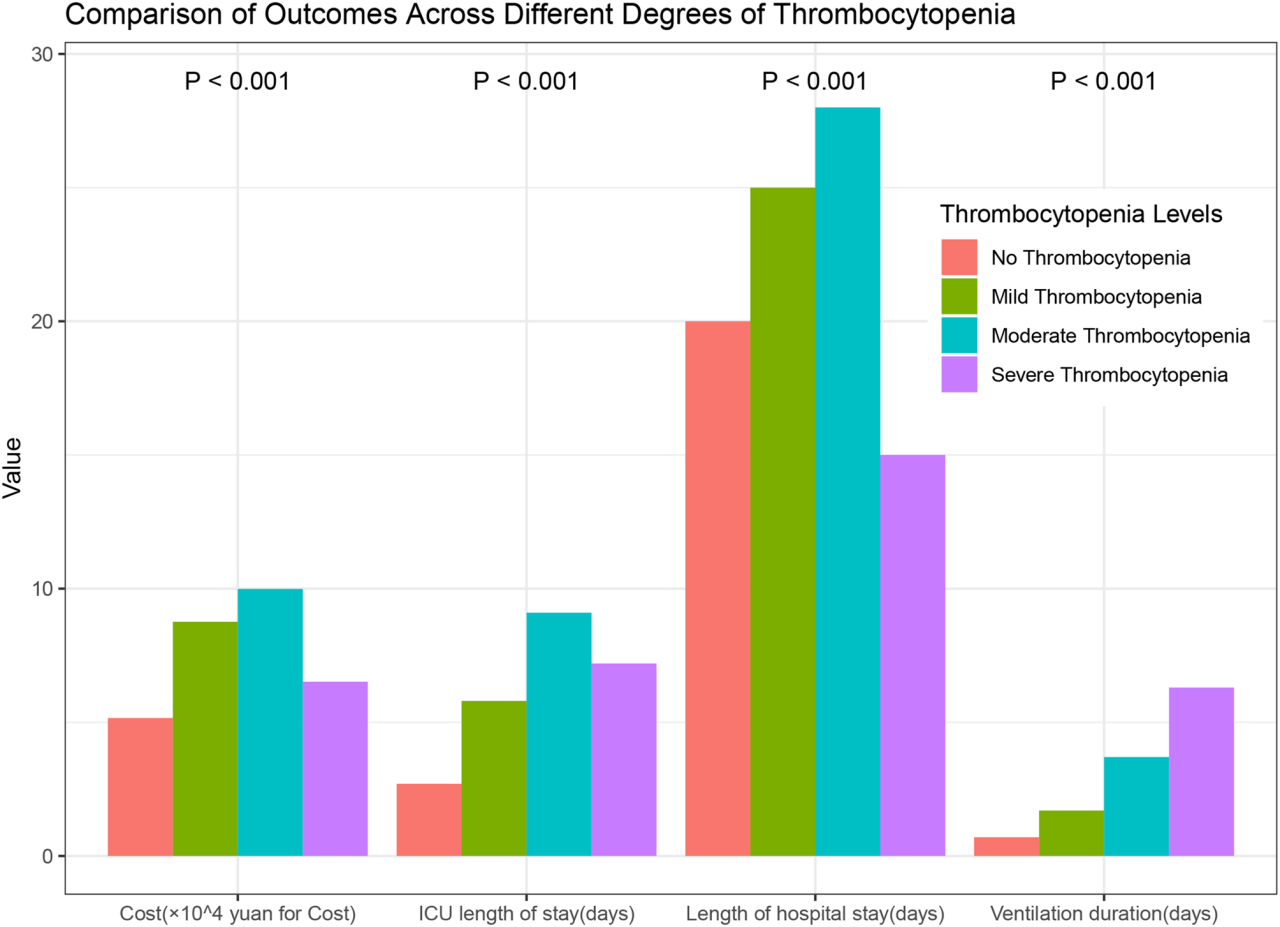
Comparison of outcomes across different degrees of thrombocytopenia.

Table 2 shows the comparison of baseline characteristics and prognostic outcomes between the thrombocytopenia and non-thrombocytopenia groups. There were statistically significant differences in age, Glasgow Coma Scale score, the proportion of hypertension cases, the proportion of long-acting glucocorticoid use, the proportion of vasoactive drug use, and the SOFA score between the thrombocytopenia and non-thrombocytopenia groups (*P* < 0.001). For biochemical indices, the thrombocytopenia group also exhibited significant differences in creatinine and hemoglobin compared to the non-thrombocytopenia group (*P* < 0.001). In terms of clinical outcomes, patients with thrombocytopenia exhibited a significantly elevated in-hospital mortality rate (22.8% compared to 8.9%, P < 0.001), reflecting poorer clinical outcomes. Moreover, with increasing severity of thrombocytopenia, a corresponding escalation in the risk of in-hospital mortality was observed, alongside prolonged mechanical ventilation, longer ICU stays, and extended hospitalization periods, resulting in higher healthcare costs (refer to Table 3, Fig. 2). Notably, while the severe thrombocytopenia group presented the highest mortality rates, it did not correspond to the longest ICU and hospital stays or the highest healthcare expenses. The incidence of thrombocytopenia in MIMIC III was 20.2%. The comparison of baseline characteristics and prognostic outcomes between the thrombocytopenia and non-thrombocytopenia groups is shown in the Additional file: Tables S2 and S3.

**Table 2:** Baseline characteristics and outcome: thrombocytopenia vs no thrombocytopenia.

Variables	No thrombocytopenia (n = 1874)	Thrombocytopenia (n = 412)	P value
Age (years), mean (SD)	76.3 ± 7.7	74.4 ± 6.7	< 0.001
Male, n (%)	989 (52.8)	235 (57)	0.129
Hypertension, n (%)	1036 (55.3)	174 (42.2)	< 0.001
Diabetes, n (%)	264 (14.1)	49 (11.9)	0.274
Surgery, n (%)			< 0.001
Cerebral	475 (25.3)	105 (25.5)	
Cardiothoracic	225 (12)	124 (30.2)	
Abdomen	457 (24.4)	102 (24.8)	
Orthopedics	353 (18.8)	29 (7.1)	
Others	364 (19.4)	51 (12.4)	
Type of glucocorticoid used			
Short acting glucocorticoids, n (%)	3 (0.2)	3 (0.7)	0.076
Intermediate glucocorticoids, n (%)	104 (5.5)	31 (7.5)	0.154
Long acting glucocorticoids, n (%)	250 (13.3)	104 (25.2)	< 0.001
Type of antiplatelet used			
Aspirin, n (%)	277 (14.8)	43 (10.4)	0.026
Tirofiban, n (%)	42 (2.2)	4 (1)	0.142
P2Y12_inhibitors, n (%)	239 (12.8)	26 (6.3)	< 0.001
Type of vasopressor used			
Dopamine, n (%)	478 (25.5)	183 (44.4)	< 0.001
Dobutamine, n (%)	91 (4.9)	42 (10.2)	< 0.001
Norepinephrine, n (%)	374 (20)	199 (48.3)	< 0.001
Adrenaline, n (%)	187 (10)	114 (27.7)	< 0.001
Glasgow coma scale, median (IQR)	15 (9, 15)	15 (7, 15)	0.038
SOFA, mean (SD)	4.5 ± 2.9	7.1 ± 2.8	< 0.001
Sepsis, n (%)	999 (53.3)	251 (60.9)	0.006
Biochemical indexes on ICU admission			
White blood cell (× 10^9^/L), mean (SD)	10.7 ± 4.5	11.2 ± 5.3	0.098
Platelet count (× 10^9^/L), mean (SD)	177.8 ± 60.5	104.3 ± 47	< 0.001
Creatinine (mmol/L), mean (SD)	76.3 ± 36.4	94.8 ± 49.9	< 0.001
Hemoglobin (g/L), mean (SD)	109.5 ± 19	102.2 ± 20.7	< 0.001
Glutamic-pyruvic transaminase (U/L), median (IQR)	17 (12, 29)	22 (14, 37)	< 0.001
Glutamic oxalacetic transaminase (U/L), median (IQR)	30 (21, 50)	52 (29, 105)	< 0.001
Prothrombin time (s), median (IQR)	14.5 (13.8, 15.5)	15.6 (14.6, 17.2)	< 0.001
Activated partial thromboplastin time (s), median (IQR)	45.7 ± 18.5	46.9 ± 17.5	0.217
C-reactive protein (mg/L), median (IQR)	57.5 (19.5, 107.2)	82.6 (42.2, 133.8)	< 0.001
Procalcitonin (ng/mL), median (IQR)	0.5 (0.1, 2.8)	1.4 (0.4, 7.1)	< 0.001
Bilirubin (μmol/L), median (IQR)	14.5 (10.3, 20.2)	16.1 (11.4, 23.8)	< 0.001
Bicarbonate (mmol/L), mean (SD)	22.1 ± 3.1	20.7 ± 3.6	< 0.001
Sodium (mmol/L), mean (SD)	139.8 ± 3.8	142 ± 4.1	< 0.001
Oxygenation index, mean ± SD	342.9 ± 113.3	330.8 ± 117.8	0.058
Vital signs, mean (SD)			
Maximum systolic pressure (mmHg)	173.4 ± 26.3	163.1 ± 26.1	< 0.001
Minimum systolic pressure (mmHg)	99 ± 16.3	89 ± 16.9	< 0.001
Maximum heartrate (bpm)	104.8 ± 20.9	112.5 ± 23.5	< 0.001
Minimum heartrate (bpm)	64.6 ± 13	68.4 ± 14.7	< 0.001
Maximum temperature (°C)	37.8 ± 0.6	37.9 ± 0.6	0.006
Minimum temperature (°C)	36.1 ± 0.7	35.8 ± 0.8	< 0.001
Outcome			
Hospital mortality, n (%)	166 (8.9)	94 (22.8)	< 0.001
ICU length of stay (days), median (IQR)	2.7 (1.8, 6.0)	6.69 (3.8, 14.0)	< 0.001
Ventilation duration (days), median (IQR)	0.7 (0.2, 2.6)	2.5 (0.8, 8.6)	< 0.001
Length of hospital stay (days), median (IQR)	20 (14, 29)	25 (15, 34)	< 0.001
Cost (× 10^3^ yuan), median (IQR)	51.55 (37.3, 77.22)	89.5 (56.2, 129.2)	< 0.001

*SOFA* sepsis-related organ failure assessment, *ICU* intensive care unit.

**Table 3:** Comparison of clinical characteristics and outcomes across different degrees of thrombocytopenia.

Variables	No thrombocytopenia (n = 1883)	Mild thrombocytopenia (n = 298)	Moderate thrombocytopenia (n = 93)	Severe thrombocytopenia (n = 24)	P value
Age (years), mean (SD)	76.3 ± 7.7	74.4 ± 6.7	74.6 ± 6.7	74.5 ± 6.1	< 0.001
Male, n (%)	992 (52.7)	170 (57)	51 (54.8)	14 (58.3)	0.513
Hypertension, n (%)	1039 (55.2)	133 (44.6)	37 (39.8)	7 (29.2)	< 0.001
Diabetes, n (%)	264 (14)	34 (11.4)	12 (12.9)	3 (12.5)	0.703
Glucocorticoid used, n (%)	345 (18.3)	98 (32.9)	29 (31.2)	8 (33.3)	< 0.001
Antiplatelet used, n (%)	307 (16.3)	43 (14.4)	6 (6.5)	1 (4.2)	0.019
Vasopressor used, n (%)	652 (34.6)	190 (63.8)	61 (65.6)	16 (66.7)	< 0.001
SOFA, mean (SD)	4.5 ± 2.9	6.6 ± 2.7	8.2 ± 2.8	8.6 ± 2.9	< 0.001
Sepsis, n (%)	1005 (53.4)	177 (59.4)	62 (66.7)	15 (62.5)	0.019
Biochemical indexes on ICU admission					
White blood cell (× 10^9^/L), mean (SD)	10.7 ± 4.5	11.3 ± 5.1	11.4 ± 5.8	9.1 ± 6.1	0.039
Platelet count (× 10^9^/L), mean (SD)	177.7 ± 60.9	111.8 ± 44	87.4 ± 52.7	92.3 ± 56.4	< 0.001
Creatinine (mmol/L), mean (SD)	76.3 ± 36.3	90.7 ± 47.2	104.6 ± 56.3	104.7 ± 48.4	< 0.001
Hemoglobin (g/L), mean (SD)	109.4 ± 19	103.9 ± 19.5	98 ± 22.2	98.8 ± 26	< 0.001
Glutamic-pyruvic transaminase (U/L), median (IQR)	17 (12, 29)	21 (14, 35)	27 (16, 47)	22.5 (13, 35.8)	< 0.001
Glutamic oxalacetic transaminase (U/L), median (IQR)	30 (21, 50)	47 (28, 99)	60 (32, 128)	48 (25.2, 89.8)	< 0.001
Prothrombin time (s), median (IQR)	14.5 (13.9, 15.4)	15.2 (14.4, 16.7)	16.3 (15.4, 18.7)	18.7 (14.7, 19.3)	< 0.001
Activated partial thromboplastin time (s), median (IQR)	45.7 ± 18.5	45.2 ± 16.5	49.8 ± 18.5	55.7 ± 20.8	0.008
Outcome					
Hospital mortality, n (%)	166 (8.8)	47 (15.8)	31 (33.3)	16 (66.7)	< 0.001
ICU length of stay (days), median (IQR)	2.7 (1.8, 6)	5.8 (3.8, 11.5)	9.1 (4.7, 17.4)	7.2 (5.2, 18.4)	< 0.001
Ventilation duration (days), median (IQR)	0.7 (0.2, 2.6)	1.7 (0.8, 6.4)	3.7 (0.7, 12.6)	6.3 (3.5, 13.1)	< 0.001
Length of hospital stay (days), median (IQR)	20 (14, 29)	25 (16, 34)	28 (16, 41)	15 (7, 27.2)	< 0.001
Cost (× 10^3^ yuan), median (IQR)	51.6 (37.2, 77.2)	87.6 (56.9, 122.6)	99.9 (61.3, 150.5)	65.2 (47.8, 130.6)	< 0.001

*SOFA* sepsis-related organ failure assessment, *ICU* intensive care unit.

### Model construction and evaluation

**Figure 3:**
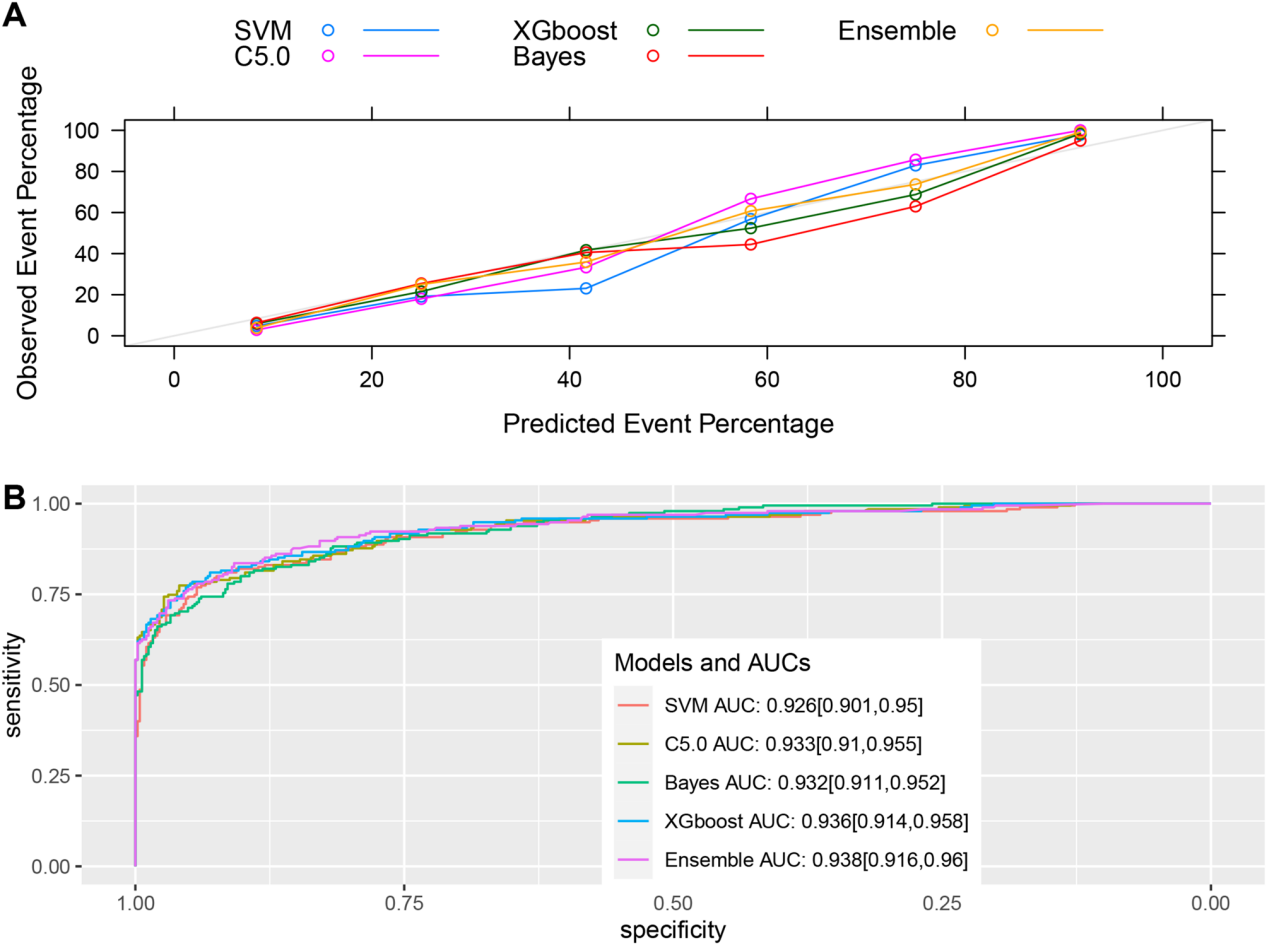
Evaluation of model performance in the internal validation dataset. (**A**) Calibration plot shows the consistency between observed and predicted risks for thrombocytopenia. (**B**) Discrimination of the machine-learning models in the internal validation dataset. *SVM* support vector machine, *XGBoost* extreme gradient boosting, *AUC* area under the curve. The number in parentheses indicates the 95% confidence interval.

**Figure 4:**
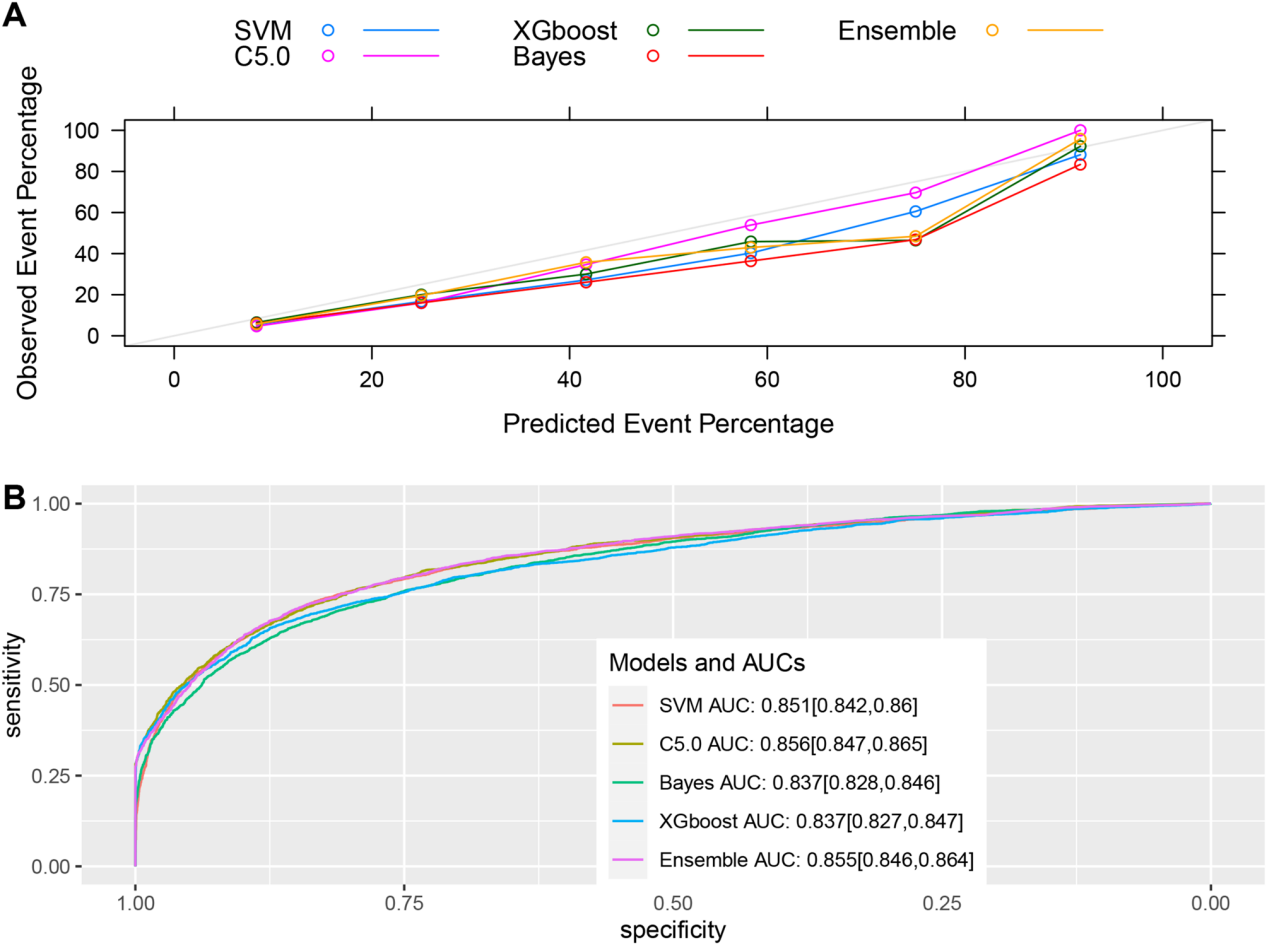
Evaluation of model performance in the external validation dataset. (**A**) Calibration plot shows the consistency between observed and predicted risks for thrombocytopenia. (**B**) Discrimination of the machine-learning models in the internal validation dataset. *SVM* support vector machine, *XGBoost* extreme gradient boosting, *AUC* area under the curve. The number in parentheses indicates the 95% confidence interval.

Models were constructed using the C5.0, SVM, Bayesian, and XGBoost ML algorithms. These four models were used to create an integrated model that fused the predictions of the four algorithms together. Figure 3 shows the receiver operating characteristic curves and calibration plots of the five different predictive models for the internal validation dataset. The AUCs for each model were approximately 0.93. Comparisons of ROC curves and model calibration plots for the external validation set are shown in Fig. 4. The model calibration plots show that all models have good predictive performance. The AUC of the C5.0 model was 0.856 (95% confidence interval [CI] 0.847–0.865), the AUC of the SVM model was 0.851 (95% CI 0.842–0.86), and the AUC of the integrated model was 0.855 (95% CI 0.846–0.864). Besides providing an AUC of 0.856, indicating the C5.0 model’s strong predictive capability, the probability cutoff that defines this curve’s specificity and sensitivity was focused on. The model demonstrated a good balance of sensitivity and specificity, with a cut-off point of 0.5, considering the applicability of the model in a clinical setting. Table 4 summarizes the predictive performance metrics of the models. For internal validation, the XGboost and Ensemble models exhibited the best accuracy (0.899), and the C5.0 model exhibited the best precision (0.978), specificity (0.924), and balanced accuracy. In external validation, the C5.0 model had the highest precision (0.98), recall (0.891), accuracy (0.884), and balanced accuracy (0.849). Overall, the C5.0 model exhibited strong predictive performance in both the internal and external validation set, especially in balanced accuracy. The predictive model for assessing the severity of thrombocytopenia demonstrated an accuracy of 0.84 with a CI of 0.81–0.86 for internal validation. For external validation using the MIMIC database, the model showed an accuracy of 0.80 with a CI of 0.79–0.81.

**Table 4:** Comparison of the additional evaluation metrics of machine learning models in internal and external validation.

Model	Accuracy	Precision	Recall	Specificity	Balanced.Accuracy
Models for predicting thrombocytopenia in internal validation					
SVM	0.892	0.943	0.909	0.842	0.876
C5.0	0.893	0.978	0.885	0.924	0.905
Bayes	0.88	0.947	0.892	0.842	0.867
XGboost	0.899	0.965	0.901	0.894	0.897
Ensemble	0.899	0.965	0.901	0.894	0.897
Models for predicting thrombocytopenia in external validation					
SVM	0.868	0.927	0.915	0.615	0.765
C5.0	0.884	0.98	0.891	0.806	0.849
Bayes	0.858	0.922	0.909	0.585	0.747
XGboost	0.879	0.959	0.902	0.706	0.804
Ensemble	0.874	0.952	0.902	0.675	0.788

*SVM* support vector machine; *XGBoost* extreme gradient boosting.

### Model explanation

**Figure 5:**
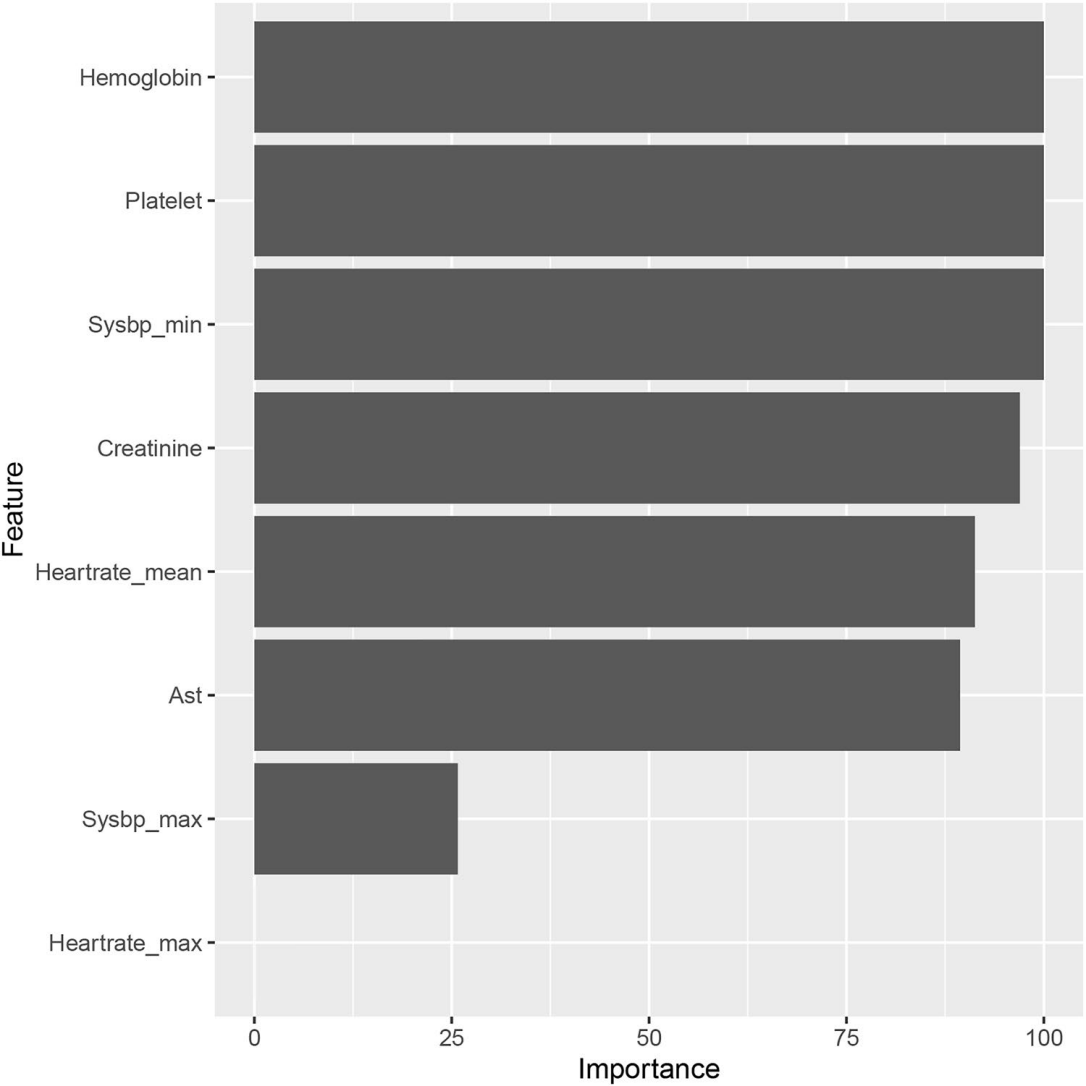
Variable-importance ranking in the C50. *AST* glutamic oxalacetic transaminase.

**Figure 6:**
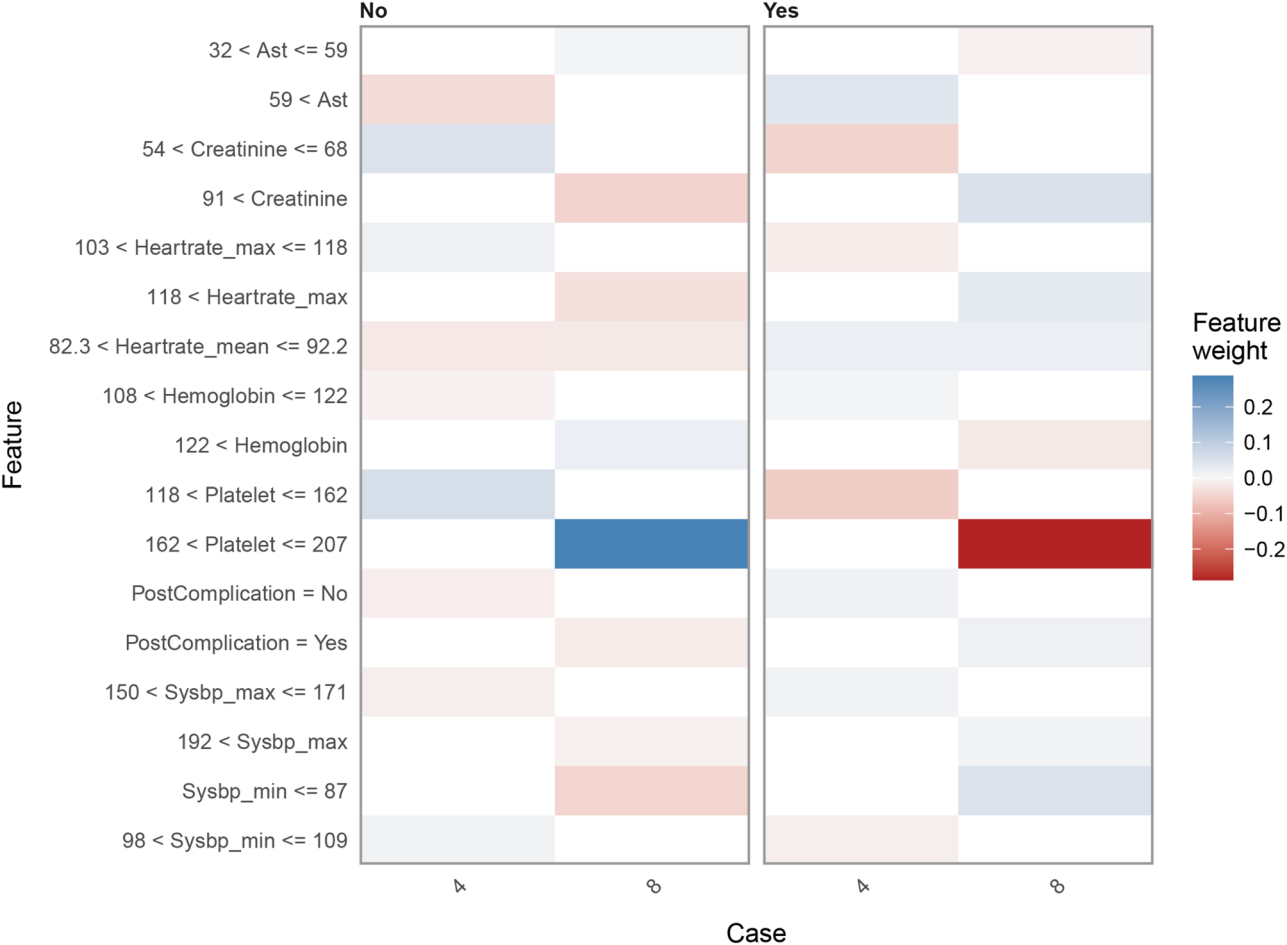
Heatmap plot showing the contribution of each variable to the classification of the sample patients. Relative contribution of each variable was calculated using the LIME algorithm. Data of patients 4 and 8 are shown as examples. Red color indicates that the relevant variable contradicts a given label, while the blue color indicates support. *AST* glutamic oxalacetic transaminase, *LIME* local interpretable model-agnostic explanations.

**Figure 7:**
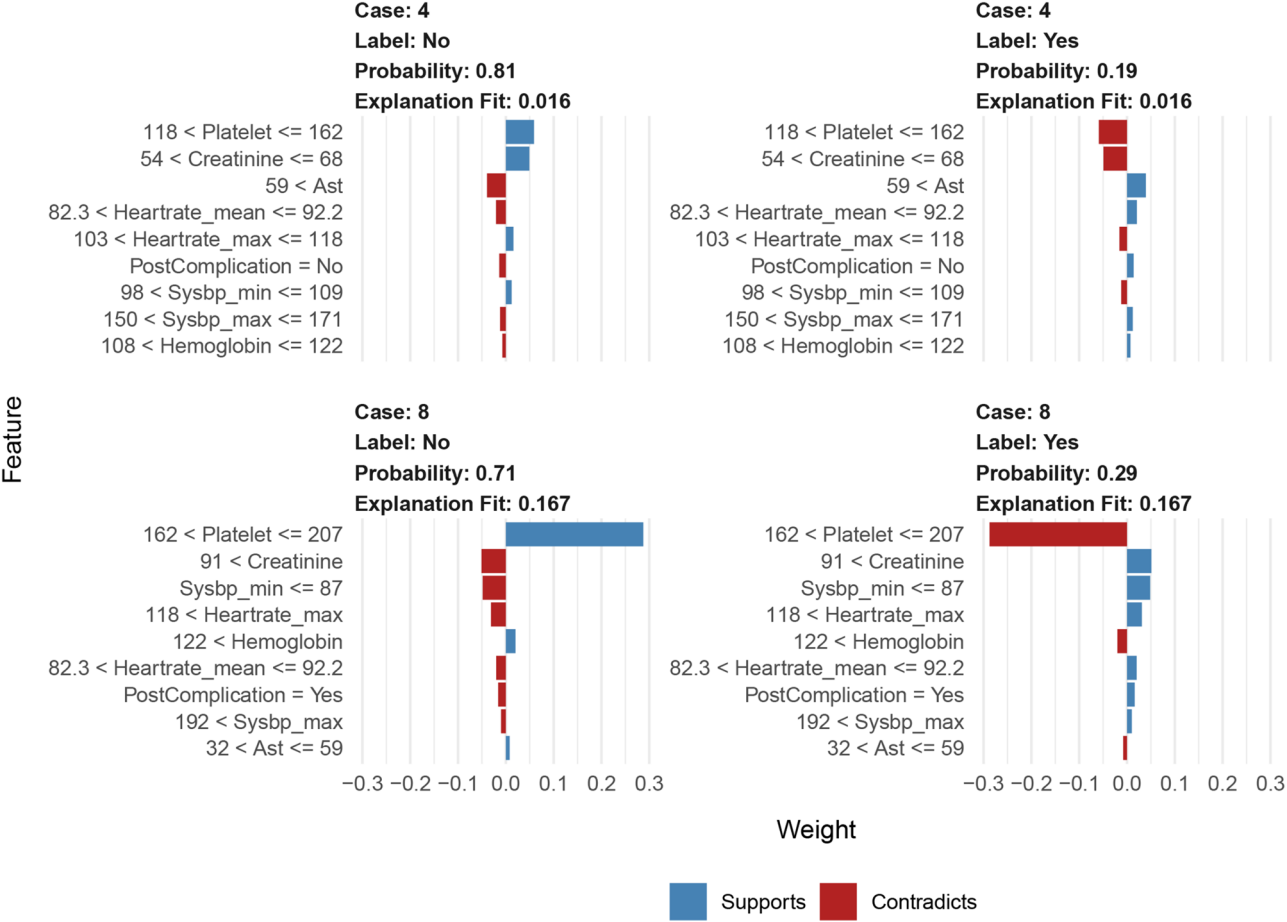
LIME feature plot shows the contribution of each variable to the classification of the sample patients. Red color indicates that the relevant variable contradicts a given label, while blue color indicates support. *AST* glutamic oxalacetic transaminase, *LIME* local interpretable model-agnostic explanations.

**Figure 8:**
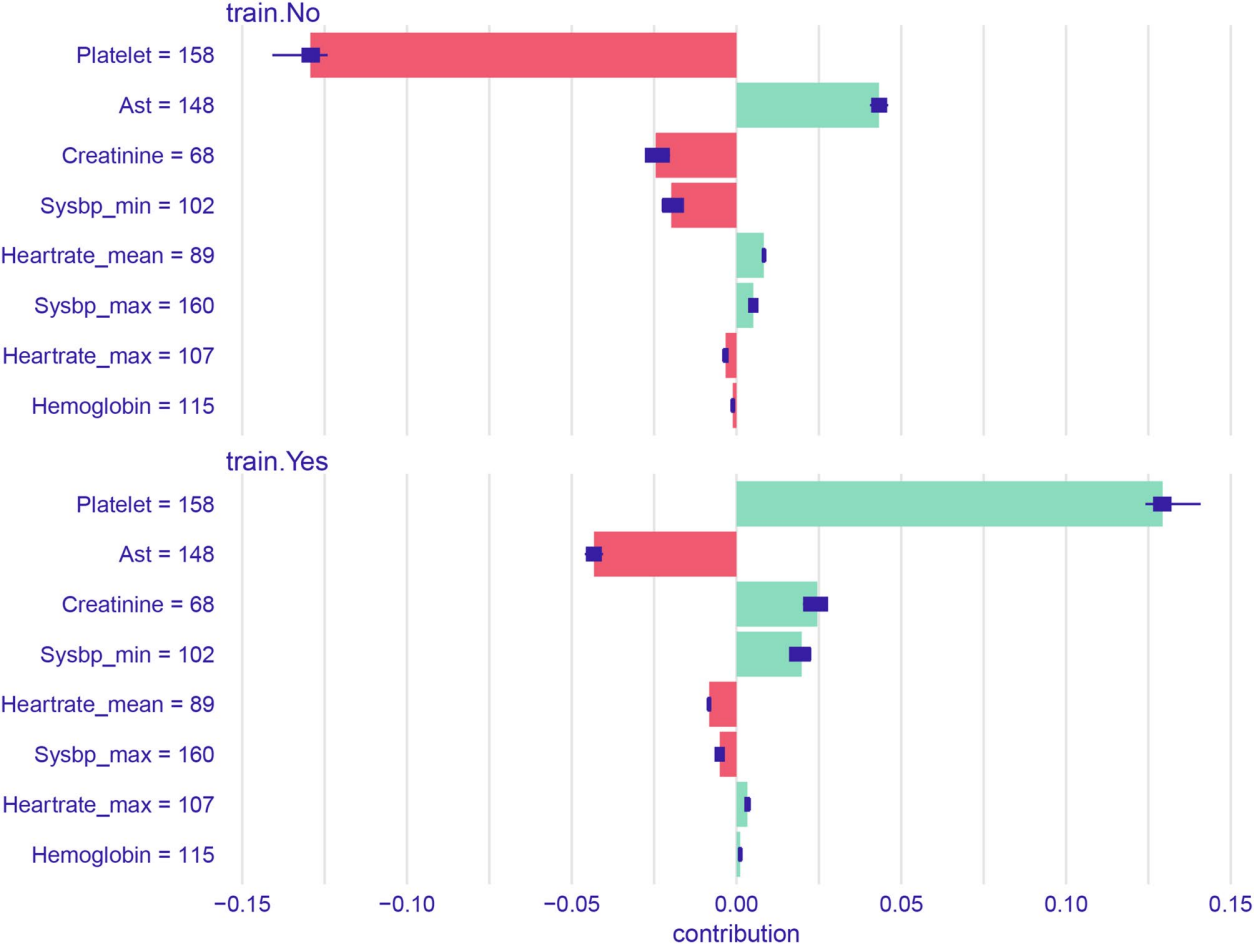
Model interpretation using the iBreakdown algorithm with uncertainty indicated by a box plot. The horizontal axis reflects the probability scale. *AST* glutamic oxalacetic transaminase.

Figure 5 shows the order of importance of the variables in the C5.0 model. Platelet count at ICU admission, hemoglobin, and minimum systolic blood pressure within 24 h of ICU admission had the largest impacts on the model. LIME provides an explanation for individual patients. Figures 6 and 7 show the variable contributions in two patients (patients 4 and 8). The contribution of each variable to the probability of developing thrombocytopenia in patient 4 was estimated using the iBreakdown algorithm (Fig. 8), which showed that initial platelet count = 158 was strongly associated with an increased risk of thrombocytopenia.

## Discussion

To our knowledge, this study is the first to use multiple machine learning algorithms in predicting the risk of thrombocytopenia in postoperative geriatric ICU patients. The results showed that the C5.0 model had an excellent ability to predict thrombocytopenia in postoperative geriatric ICU patients, and its AUC, accuracy, precision, specificity, and recall were satisfactory, thus providing reliable reference for clinicians.

Our research showed that the extent of platelet drop was directly correlated with the disease severity. In other words, the more severe the reduction in platelets, the higher the patient mortality rate, the longer the hospital stay, and the higher the hospitalization costs. This aligns with previous research findings, suggesting that the severity of platelet decrease can assist in the early identification of patient prognosis^24^. It should also be noted that sepsis is a major cause of thrombocytopenia in the ICU^25^. Our study revealed that there was a higher incidence of sepsis in those with decreased platelets. Common inflammation indicators such as C-reactive protein and procalcitonin levels were higher, making patients with severe sepsis more susceptible to platelet decrease.

The potential for machine learning algorithms to process large amounts of data and capture complex patterns to provide more accurate predictions becomes apparent during the model construction process. Similar efforts in the field of medical research confirm our approach. Maray et al*.* developed a machine learning-based predictive model for early identification of linezolid-induced thrombocytopenia based on a database of critical care medical data. The model had good predictive performance, with accuracy, sensitivity, specificity, and AUC of 0.75, 0.78, 0.62, and 0.80, respectively^26^. In the present study, several algorithms were evaluated, and an integrated algorithm was used, ultimately finding that the C5.0 model had the best prediction performance and explanation. This may be due to the decision tree structure of the C5.0 model, which enables visual representation of the relationships in the data, making the model not only highly accurate but also easy to interpret and widely applicable to medical data^27,28^. Additionally, the SVM is one of the best techniques in recent years for dividing data into multiple categories and is widely used for classification problems. In our study, the SVM showed good predictive performance in the model predicting the severity of thrombocytopenia^29^.

Notably, several key predictor variables were identified, namely aspartate transaminase, creatinine, hemoglobin, systolic blood pressure, and heart rate, which are all common clinical parameters that are easy to collect. Thrombocytopenia is common in patients with acute kidney injury (AKI) and has been shown to be a significant predictor of mortality. A recent study systematically evaluating AKI and platelet function demonstrated the predictive value of creatinine for thrombocytopenia^30^. In addition, liver function is closely associated with platelets, and thrombocytopenia is common in patients with cirrhosis and chronic liver disease. Studies have shown that aspartate transaminase is predictive of chemotherapy-induced thrombocytopenia in patients with cancer^31^.

This study has several limitations. First, this study was conducted based on retrospective data with some missing data. Second, the causes of thrombocytopenia in critically ill patients are diverse and primarily include increased energy consumption, hemodilution, infections, medication, and immune-related factors^32,33^. Prominent factors leading to drug-induced thrombocytopenia, such as HIT, were excluded. Regardless, the reliable exclusion of all cases of drug-induced thrombocytopenia remains challenging despite their low representation in the ICU^34^. Finally, a model for specifically predicting thrombocytopenia after ICU admission was constructed. In patients with a prolonged duration of ICU stay, the ability to predict the risk of thrombocytopenia in the next 24 or 48 h may be valuable. This initiative aligns with our current endeavors to integrate this predictive model into advanced electronic health record systems for prompt results and early intervention.

Despite the limitations, the strengths of this study include the use of various machine learning algorithms and detailed model evaluation and validation to ensure the robustness and reliability of the models. Moreover, the explanation of the model was further enhanced using algorithms, such as LIME and iBreakdown, and a web calculator based on the C5.0 model was developed, which enhances the explanation of the model for clinical applications. Finally, the data used for model construction are from hospitals in China, and the external validation data are from the MIMIC dataset from the United States. Due to differences in culture, healthcare systems, and baseline characteristics of patients, the predictive validity of our model was measured by multiple parameters, especially balanced accuracy, with the potential for broader geographic applicability.

## Conclusion

This study furnishes valid, reliable, and easily interpretable predictive models for assessing the risk and the severity of thrombocytopenia in postoperative geriatric ICU patients. These models are anticipated to provide clinicians with robust tools to aid in decision-making with regard to patient care. Further research, along with prospective validation, is welcomed to refine and expand the potential applications of these models.

### Supplementary Information

Supplementary Information.

## Data Availability

The datasets of Dongyang People’s Hospital are available from the corresponding author on reasonable request. The MIMIC III dataset is a freely accessible online critical care database (https://mimic.physionet.org/). The main code and web calculator mentioned in the article can be accessed at https://github.com/fzs1412/Predicting-Platelet-Drop-in-ICU-Elderly.git.
