# Supplementary material for: Retrospective analysis of interpretable machine learning in predicting ICU thrombocytopenia in geriatric ICU patients
Source: Sci Rep. 2024 Jul 20;14:16738. doi: 10.1038/s41598-024-67785-1 (PMC11271295; doi:10.1038/s41598-024-67785-1)
Supplement: Supplementary file 1 — Supplementary Information. [file 41598_2024_67785_MOESM1_ESM.pdf]

Table S1 Transparent Reporting of a multivariable prediction model for Individual Prognosis or Diagnosis Statement

Section/Topic	Item		Checklist Item	Page
**Title and abstract**				
Title	1	D;V	Identify the study as developing and/or validating a multivariable prediction model, the target population, and the outcome to be predicted.	Title
Abstract	2	D;V	Provide a summary of objectives, study design, setting, participants, sample size, predictors, outcome, statistical analysis, results, and conclusions.	Abstract
**Introduction**				
Background and objectives	3a	D;V	Explain the medical context (including whether diagnostic or prognostic) and rationale for developing or validating the multivariable prediction model, including references to existing models.	Introduction
	3b	D;V	Specify the objectives, including whether the study describes the development or validation of the model or both.	Introduction
**Methods**				
Source of data	4a	D;V	Describe the study design or source of data (e.g., randomized trial, cohort, or registry data), separately for the development and validation data sets, if applicable.	Methods, Study design
	4b	D;V	Specify the key study dates, including start of accrual; end of accrual; and, if applicable, end of follow-up.	Methods, Study Design
Participants	5a	D;V	Specify key elements of the study setting (e.g., primary care, secondary care, general population) including number and location of centres.	Methods, Study Design
	5b	D;V	Describe eligibility criteria for participants.	Methods, Study Design
	5c	D;V	Give details of treatments received, if relevant.	Not applicable
Outcome	6a	D;V	Clearly define the outcome that is predicted by the prediction model, including how and when assessed.	Methods, Data collection
	6b	D;V	Report any actions to blind assessment of the outcome to be predicted.	Not applicable
Predictors	7a	D;V	Clearly define all predictors used in developing the multivariable prediction model, including how and when they were measured.	Methods, Data collection
	7b	D;V	Report any actions to blind assessment of predictors for the outcome and other predictors.	Not applicable
Sample size	8	D;V	Explain how the study size was arrived at.	Not applicable
Missing data	9	D;V	Describe how missing data were handled (e.g., complete-case analysis, single imputation, multiple imputation) with details of any imputation method.	Methods, Data processing
Statistical analysis methods	10a	D	Describe how predictors were handled in the analyses.	Methods, Statistical analysis
	10b	D	Specify type of model, all model-building procedures (including any predictor selection), and method for internal validation.	Methods, Model construction
	10c	V	For validation, describe how the predictions were calculated.	Methods, Model evaluation
	10d	D;V	Specify all measures used to assess model performance and, if relevant, to compare multiple models.	Methods, Model evaluation
	10e	V	Describe any model updating (e.g., recalibration) arising from the validation, if done.	Not applicable
Risk groups	11	D;V	Provide details on how risk groups were created, if done.	Not applicable
Development vs. validation	12	V	For validation, identify any differences from the development data in setting, eligibility criteria, outcome, and predictors.	Methods, Model evaluation
Results				
Participants	13a	D;V	Describe the flow of participants through the study, including the number of participants with and without the outcome and, if applicable, a summary of the follow-up time. A diagram may be helpful.	Results, Figure 1
	13b	D;V	Describe the characteristics of the participants (basic demographics, clinical features, available predictors), including the number of participants with missing data for predictors and outcome.	Results, Table 2
	13c	V	For validation, show a comparison with the development data of the distribution of important variables (demographics, predictors and outcome).	Results, Additional file
Model development	14a	D	Specify the number of participants and outcome events in each analysis.	Results
	14b	D	If done, report the unadjusted association between each candidate predictor and outcome.	Not applicable
Model specification	15a	D	Present the full prediction model to allow predictions for individuals (i.e., all regression coefficients, and model intercept or baseline survival at a given time point).	Results, Model explanation
	15b	D	Explain how to use the prediction model.	Results, Additional file
Model performance	16	D;V	Report performance measures (with CIs) for the prediction model.	Results, Table 3
Model-updating	17	V	If done, report the results from any model updating (i.e., model specification, model performance).	Not applicable
**Discussion**				
Limitations	18	D;V	Discuss any limitations of the study (such as nonrepresentative sample, few events per predictor, missing data).	Discussion Limitations
Interpretation	19a	V	For validation, discuss the results with reference to performance in the development data, and any other validation data.	Discussion
	19b	D;V	Give an overall interpretation of the results, considering objectives, limitations, results from similar studies, and other relevant evidence.	Discussion, Conclusion
Implications	20	D;V	Discuss the potential clinical use of the model and implications for future research.	Conclusion
**Other information**				
Additional information	21	D;V	Provide information about the availability of Additional resources, such as study protocol, Web calculator, and data sets.	Additional file
Funding	22	D;V	Give the source of funding and the role of the funders for the present study.	Declarations Funding

*Items relevant only to the development of a prediction model are denoted by D, items relating solely to a validation of a prediction model are denoted by V, and items relating to both are denoted D;V. We recommend using the TRIPOD Checklist in conjunction with the TRIPOD Explanation and Elaboration document.

Table S2 Comparisons of Baseline Characteristics and Outcome Between with Thrombocytopenia and No Thrombocytopenia in MIMIC III

Variables	No Thrombocytopenia (n = 5708)	Thrombocytopenia (n = 1443)	P value
Age (years), mean (SD)	76.8 ± 7.3	77.1 ± 6.6	0.205
Gender, n (%)	3118 (54.6)	825 (57.2)	0.088
Hypertension, n (%)	3479 (60.9)	809 (56.1)	< 0.001
Diabetes, n (%)	1637 (28.7)	387 (26.8)	0.171
Ethnicity, n (%)			< 0.001
ASIAN	101 (1.8)	28 (1.9)	
BLACK	187 (3.3)	37 (2.6)	
HISPANIC	89 (1.6)	22 (1.5)	
OTHER	942 (16.5)	312 (21.6)	
WHITE	4389 (76.9)	1044 (72.3)	
SOFA, mean (SD)	3.8 ± 2.3	6.2 ± 2.5	< 0.001
Glucocorticoid used, n (%)	297 (5.2)	82 (5.7)	0.509
Vasopressor used, n (%)	753 (13.2)	547 (37.9)	< 0.001
RRT, n (%)	30 (0.5)	84 (5.8)	< 0.001
Biochemical indexes on ICU admission			
Red blood cell (x109/L), mean (SD)	3.5 ± 0.6	3.2 ± 0.6	< 0.001
White blood cell (x109/L), mean (SD)	12.1 ± 4.6	11.6 ± 5	< 0.001
Creatinine (mmol/L), median (IQR)	79.6 (61.9, 97.2)	79.6 (61.9, 106.1)	0.022
Platelet count(x109/L), mean (SD)	214.2 ± 76.8	127.8 ± 51.6	< 0.001
Lactate (mmol/L), median (IQR)	1.5 (1.1, 2.2)	1.8 (1.2, 3)	< 0.001
pH, mean (SD)	7.4 ± 0.1	7.4 ± 0.1	0.003
Glutamic oxalacetic transaminase (U/L), mean (SD)	61 ± 74.2	86.1 ± 96.6	< 0.001
Vital signs, mean (SD)			
Minimum systolic pressure (mmHg)	91 ± 15.6	83.4 ± 14.4	< 0.001
Maximum systolic pressure (mmHg)	155.4 ± 20.7	152.8 ± 20.2	< 0.001
Minimum diastolic pressure (mmHg)	41.2 ± 8.4	39.9 ± 8.2	< 0.001
Maximum diastolic pressure (mmHg)	80.3 ± 15.3	76.6 ± 13.6	< 0.001
Outcome			
ICU length of stay (days), median (IQR)	2.3 (1.4, 4.2)	3.6 (2.1, 7.9)	< 0.001
Length of hospital stay (days) , median (IQR)	7.83 (5.3, 12.0)	9.9 (6.9, 16.3)	< 0.001
Ventilation duration (days), median (IQR)	4.93 (0, 18)	16 (5, 51.3)	< 0.001

Hospital mortality, n (%)	621 (10.9)	243 (16.8)	< 0.001

SOFA: Sepsis-related Organ Failure Assessment; RRT: Renal replacement therapy.

Table S3 Clinical Characteristics and Outcomes by Thrombocytopenia Severity in MIMIC III

Variables	No Thrombocytopenia (n = 5708)	Mild Thrombocytopenia (n = 244)	Moderate Thrombocytopenia (n = 1052)	Severe Thrombocytopenia (n = 147)	P value
Age (years), mean (SD)	76.8 ± 7.3	77.1 ± 6.5	77 ± 6.7	77.4 ± 6.6	0.613
Gender, n (%)	3118 (54.6)	151 (61.9)	606 (57.6)	68 (46.3)	0.006
SOFA, mean (SD)	3.8 ± 2.3	5.3 ± 2.4	6.2 ± 2.4	7.7 ± 2.3	< 0.001
Hypertension, n (%)	3479 (60.9)	145 (59.4)	597 (56.7)	67 (45.6)	< 0.001
Diabetes, n (%)	1637 (28.7)	67 (27.5)	291 (27.7)	29 (19.7)	0.11
Vasopressor used, n (%)	753 (13.2)	59 (24.2)	398 (37.8)	90 (61.2)	< 0.001
Biochemical indexes on ICU admission					
Platelet count(x109/L), mean (SD)	214.2 ± 76.8	148.8 ± 60.2	123.1 ± 46.6	126.1 ± 61.2	< 0.001
Hemoglobin (g/L), mean (SD)	10.6 ± 1.8	10.2 ± 1.8	9.9 ± 1.8	9.9 ± 2	< 0.001
Creatinine (mmol/L) , mean (SD)	79.6 (61.9, 97.2)	79.6 (61.9, 106.1)	79.6 (61.9, 106.1)	97.2 (61.9, 132.6)	< 0.001
Glutamic oxalacetic transaminase (U/L), mean (SD)	61 ± 74.2	79 ± 91.9	82.8 ± 93.9	121.4 ± 115	< 0.001
Outcome					
ICU length of stay (days), median (IQR)	2.3 (1.4, 4.2)	2 (1.2, 4.1)	3.9 (2.2, 7.3)	9 (4.3, 15.2)	< 0.001
Length of hospital stay (days) , median	4.9 (0, 18)	7.4 (2.9, 19.3)	16.2 (5.3, 49.5)	45.9 (16.1, 142)	< 0.001

(IQR)					
Ventilation duration (days), median (IQR)	7.8 (5.2, 12)	8.2 (6.2, 13.8)	9.9 (6.9, 15.6)	15.2 (9.2, 26.1)	< 0.001
Hospital mortality, n (%)	621 (10.9)	48 (19.7)	133 (12.6)	62 (42.2)	< 0.001

SOFA: Sepsis-related Organ Failure Assessment; RRT: Renal replacement therapy.

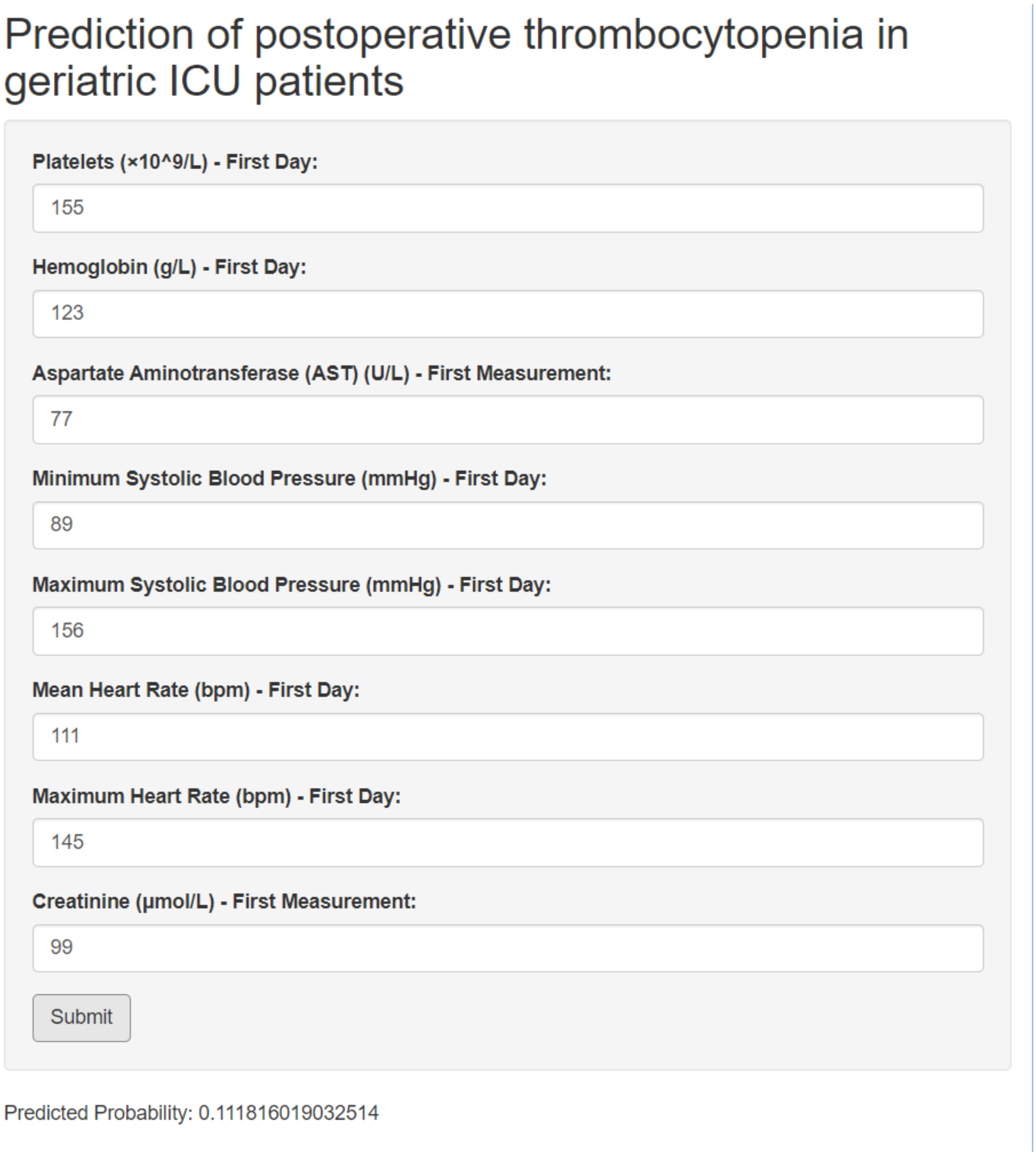

Figure S1 ICU thrombocytopenia calculator based on the C5.0 algorithm model

Abbreviations: ICU: Intensive Care Unit; AST: glutamic oxalacetic transaminase.

Instructions: To use the web calculator, please visit our GitHub page (https://github.com/fzs1412/Predicting-Platelet-Drop-in-ICU-Elderly.git). Download the repository and load the "C50_model.rds " file using R software. This tool allows you to calculate the probability of thrombocytopenia in ICU patients by inputting the necessary clinical parameters.

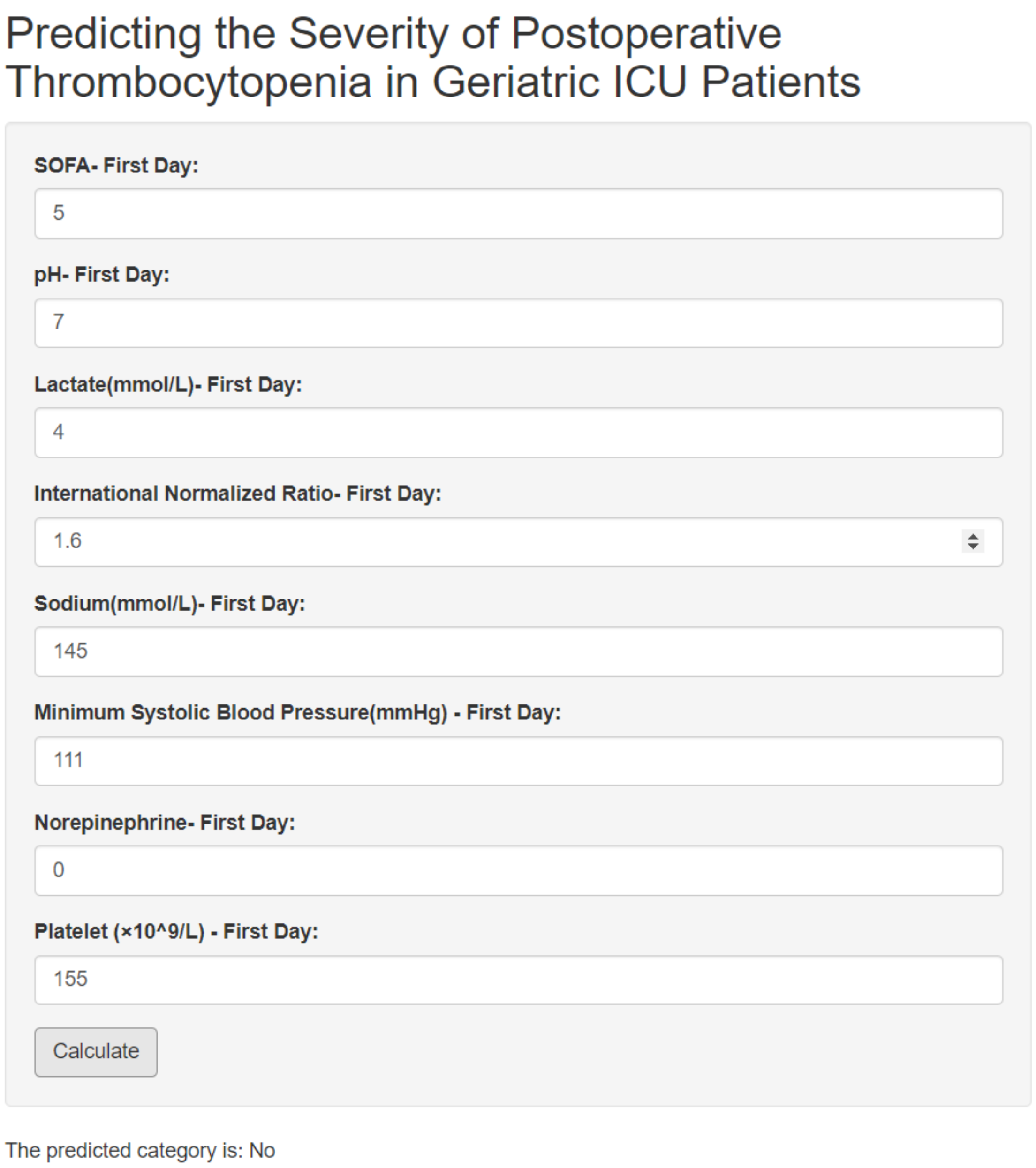

Figure S2 Predicting the Severity of Postoperative Thrombocytopenia in Geriatric ICU Patients based on the SVM algorithm model

Abbreviations: ICU: Intensive Care Unit; SOFA: Sepsis-related Organ Failure Assessment.

Instructions: To use the web calculator, please visit our GitHub page (https://github.com/fzs1412/Predicting-Platelet-Drop-in-ICU-Elderly.git). Download the repository and load the "svmFit_model.rds " file using R software. This tool allows you to calculate the severity of thrombocytopenia in ICU patients by inputting the necessary clinical parameters.
